# Supplementary material for: The genome sequence of the anthomyzid fly, Anthomyza gracilis Fallén, 1823 (Diptera: Anthomyzidae)
Source: Wellcome Open Res. 2025 Nov 21;10:649. [Version 1] doi: 10.12688/wellcomeopenres.25281.1 (PMC12759283; doi:10.12688/wellcomeopenres.25281.1)
Supplement: Peer review history

# Reviewer report: Reviewer response for version 1

**DOI:** 10.21956/wellcomeopenres.27862.r141604

**Published:** 2026-01-02

**Authors:** Kristina Gagalova

**Affiliations:** 1 Curtin University, Perth, Australia

Thank you for submitting the genome assembly of *Anthomyza gracilis. *Overall, I found the manuscript to be clear, and the methods well described. I only have a few suggestions/comments:

 1) Could you please provide some more background info about the species? Why did you choose it for sequencing? Why a female individual?

 2) You mention that the specimen was stored in ethanol and the DNA is extracted from a small specimen. Is there any further recommendation on how to process this sample for HMW DNA? Can you provide more details?

 3) What is the purpose of dual PCR? Could you please specify its motivation? Have you evaluated if they both worked effectively?

 4) What ploidy did you assume when running GenomeScope? Do you have evidence for diploidy from other data (flow cytometry or karyotyping)?

 5) Cold you please add a separate paragraph for the curation of the sex chromosome?

 6)  What fraction of the assembled genome corresponds to bacterial or other biological contaminants? Have you assessed the presence of bacterial or fungal symbionts? Contamination screening can provide valuable biological insight, as assembled non-host sequences sometimes reveal previously uncharacterized species or co-existing microbial communities. Reporting these findings, where present, would strengthen the study by clarifying genome purity while also highlighting potential host–microbe associations.

Are sufficient details of methods and materials provided to allow replication by others?

Yes

Is the rationale for creating the dataset(s) clearly described?

Yes

Are the datasets clearly presented in a useable and accessible format?

Yes

Are the protocols appropriate and is the work technically sound?

Yes

Reviewer Expertise:

plant and insect genomics, pangenomics, comparative genomics, phylogenomic, genome sequencing, structural bioinformatics, fungal genomics

I confirm that I have read this submission and believe that I have an appropriate level of expertise to confirm that it is of an acceptable scientific standard.

# Reviewer report: Reviewer response for version 1

**DOI:** 10.21956/wellcomeopenres.27862.r140751

**Published:** 2026-01-02

**Authors:** Stuart J.E. Baird

**Affiliations:** 1 Academy of Sciences of the Czech Republic, Národní, Staré Město, Czech Republic

The manuscript conforms to the excellent DToL template. I find no typos, no formatting issues. It could perhaps be explained better why it was necessary for a second round of DNA damage repair to be applied on the pooled DNA. It is regrettable that there is no identification of sex chromosome(s), but apparently unavoidable.

Are sufficient details of methods and materials provided to allow replication by others?

Yes

Is the rationale for creating the dataset(s) clearly described?

Yes

Are the datasets clearly presented in a useable and accessible format?

Yes

Are the protocols appropriate and is the work technically sound?

Yes

Reviewer Expertise:

Admixture genomics. Spatial genetics.

I confirm that I have read this submission and believe that I have an appropriate level of expertise to confirm that it is of an acceptable scientific standard.
